# Multimodal and multifunctional signaling? – Web reduction courtship behavior in a North American population of the false black widow spider

**DOI:** 10.1371/journal.pone.0228988

**Published:** 2020-02-26

**Authors:** Andreas Fischer, Xiang Hao Goh, Jamie-Lynne S. Varney, Adam J. Blake, Stephen Takács, Gerhard Gries

**Affiliations:** Department of Biological Sciences, Simon Fraser University, Burnaby, BC, Canada; Universidade de São paulo, BRAZIL

## Abstract

Males of widow spiders courting on the web of females engage in web-reduction behavior which entails excising a section of the web, bundling it up, and wrapping it with their silk. Males of the false black widow spider, *Steatoda grossa*, in European populations also produce stridulatory courtship sound which has not yet been studied in their invaded North American range. Working with a North American population of *S*. *grossa*, we tested the hypotheses that (1) web reduction by males renders webs less attractive to rival males; (2) deposition of silk by courting males has an inter-sexual (male-female) signal function that enhances their likelihood of copulation; and (3) stridulatory sound is a courtship signal of males. Testing anemotactic attraction of males in Y-tube olfactometer experiments revealed that reduced webs (indicative of a mated female) and intact webs (indicative of a virgin female) were equally attractive to males. Recording courtship behavior of males with either functional (silk-releasing) spinnerets or spinnerets experimentally occluded on the web of virgin females showed that males with functional spinnerets were more likely to copulate with the female they courted. Although males possess the stridulatory apparatus to produce courtship sound, they did not stridulate when courting or copulating on the web of females. Our data support the conclusion that web-reduction behavior of *S*. *grossa* males in their invaded North American range has no long-range effect on mate seeking males. Instead, web-reduction behavior has an inter-sexual signaling function that seems to be linked to functional spinnerets of the courting male. The signal produced by a male likely entails a volatile silk-borne pheromone, but may also embody a gauge of his endurance (the amount of time he engages in web reduction causing web vibrations).

## Introduction

During courtship, many animals produce multi-modal signals that function between prospective mates. These inter-sexual (male-female) signals (*i*) offer information about the signaler including sex, age, dominance and health [1–4], (*ii*) reduce aggression between partners [5], and (*iii*) render the female receptive to the male [6]. Courting males display diverse signals of one or more sensory modalities. Males of birds of paradise, e.g., use visual signals “showing off” their extraordinary plumage [7], male crickets stridulate producing sound [8], male tiger moths emit pheromones [9], and males of web-building spiders vibrate the female’s web [10].

Concurrently, one or both sexes may also produce intra-sexual signals that deter potential rivals [11,12]. Inter- and intra-sexual signals can be identical [11,13] or different [14–16]. For example, the boatwhistle vocalizations of male Lusitanian toadfish, *Halobatrachus didactylus*, have dual functions, serving a role during male-female courtship and as a male-male territorial signal [11].

Courtship signals may be adjusted according to the environmental setting. In the increasingly noisy urban “soundscape”, birds upshift frequency components of their songs thus improving the apparency of their signals [17,18]. Urban habitats especially can create reproductive isolation barriers and thus genetic bottlenecks in various taxa [19–22]. Sexual selection pressure modulates courtship behavior including the honesty of sexual communication signals [23,24]. Males of the Hermann’s tortoise, *Testudo hermanni hermanni*, engage in courtship that enables a female to assess their condition [25].

Courtship signals with multiple components and modalities (e.g., courting males exhibiting visual displays, emitting pheromone or sound, generating substrate-borne vibrations, all concurrently) offer rich opportunities to investigate how courted females integrate this complex information and use it to select mates [26]. Two main hypotheses have been proposed for the evolution of such multi-modal sexual signals: (1) different signals, or signal modalities, each convey different information (the ‘multiple message’ hypothesis) and (2) different signals convey the same information (the ‘backup message’ hypothesis) [26]. By experimentally suppressing one or more signal modalities of the courting male, and by studying the behavioral responses of courted females and rival males, the information content, relative importance and the intended recipient of each signal modality can be deduced.

Web-building spiders are potential model organisms to study the specific function(s) of multimodal courtship signals. For example, males of the western black widow spider, *Latrodectus hesperus*, court a female by cutting sections of her web (potential vibratory signals) and bundling them with their own silk (potential pheromonal signal) [27]. Either signal, or the combined effect of both signals, renders the female receptive and decreases the likelihood of aggression towards the male [5,27,28]. Web reduction may also reduce the attractiveness of the female’s web to rival males [13]. However, the underlying mechanisms for the decreased attractiveness of reduced webs are not well understood. Bundled-up and compacted sections of a female’s web have reduced surface area and thus are thought to curtail pheromone dissemination [29]. Also, the male’s silk may block emanation of female pheromone from bundled web sections and/or may release a pheromone deterrent to other males [30].

Courtship signals of the closely related [31] false black widow spider, *Steatoda grossa*, seem even more complex and thus worthy of study. Males of a North American population engage in web-reduction behavior resembling that of *L*. *hesperus* [32–34], whereas *S*. *grossa* males in Europe produce audible stridulatory courtship sound (1 kHz and 3–7 kHz) (see S1 Fig) [34–38] by abdominal up- and down movements (0.008 s and 0.005 s, respectively) causing teeth-like structures on the abdomen to scrape over ridges on the cephalothorax (prosoma) [38]. the resulting stridulatory sound is thought to have both a male-male and a male-female signal function [39,40]. Moreover, two recent studies on *S*. *grossa* in Europe noted both stridulatory courtship sound and web-reduction behavior by males [33,34], suggesting an intricate interplay of sound, vibratory and pheromonal courtship signals conveyed by males. Whether males of *S*. *grossa* in North America, following the introduction of *S*. *grossa* to the New World early in the last century [41], also produce courtship sound has yet to be studied. Chemical communication, in contrast, is well documented. Silk of virgin females, and methanol extract thereof, both trigger web reduction and silk deposition by males [32]. Observations that males, engaging or not in web-reduction behavior during pre-copulatory courtship, deposit silk on the female’s web [32], imply–but not experimentally prove–a sexual communication function of male silk or silk-borne pheromone. The male’s multi-modal courtship also includes vibratory elements such as abdomen vibrations, pulling web strings with one or more appendages, front-leg drumming, and drumming on the female’s 4^th^ leg pair [32,34,38].

Courting *S*. *grossa* males exhibit two prominent behavioral elements: (*i*) web reduction (vibratory signals) with silk deposition (potential pheromonal signal), and (*ii*) abdomen vibrations (vibratory signal and potential auditory signal). This paper aims to study the specific functions and signaling modalities of these courtship behaviors by assessing their effects on female aggression towards males, copulatory success of males, and curtailed male competition. Working with a North American population of *S*. *grossa*, we tested the hypotheses that (1) web reduction by males renders webs less attractive to rival males; (2) deposition of silk by courting males has an inter-sexual signal function that enhances their likelihood of copulation; and (3) stridulatory sound is a courtship signal of males.

## Methods

### Model organism

Female *S*. *grossa* build their cob-webs in dry and warm places, often within buildings. Females and males live up to 6 and 1.5 years, respectively [38,42]. Males are polygynous and females are polyandrous with first sperm precedence [32,34,38], as reported in *L*. *hesperus*. Unlike *Latrodectus* males, mature *S*. *grossa* males build webs for prey-capture [38]. Female *S*. *grossa* have been observed to cannibalize males during copulation [33].

### Experimental spiders

Experimental spiders were the F1 generation offspring of 182 mated females collected in hallways of Simon Fraser University [43]. Two weeks after juveniles hatched (from many different cocoons), they were separated and kept singly in Petri dishes (100 × 20 mm) and provisioned once a week with the vinegar flies *Drosophila melanogaster*. Sub-adult males and sub-adult females were kept in separate rooms to prevent males from undergoing accelerated maturation [44,45]. Once a week, sub-adult and adult females and males were fed larvae of the beetle *Tenebrio molitor* and adult black blow flies, *Phormia regina*, respectively. Adult males were kept in petri dishes (100 × 200 mm), whereas adult females were kept in 300-ml clear plastic cups (Western Family, Tigard, OR, USA). All spiders had access to water in cotton wicks re-moistened once a week. Spiders were maintained at 22°C under a reversed photoperiod (12 h:12 h). All experiments were run during the scotophase. Only mature males (>7 days post final moult) and mature virgin females (>10 days post final moult) [30] were tested in experiments. Male-female pairs in courtship trials were not siblings.

### H1: Web reduction by males renders webs less to rival males

Hypothesis 1 was tested in Y-tube Pyrex glass olfactometer experiments. For each experimental replicate, a male was introduced into a glass “holding” tube (2 × 26 cm) and allowed 2 min to acclimatize before the tube was connected via a glass joint to the Y-tube olfactometer (main stem: 24 cm, side arms: 21 cm, diam: 2.5 cm) [46]. A translucent oven bag (30 × 31 cm, Toppits, Mengen, Germany) containing a test stimulus such as a female web was secured to the orifice of each side arm. The opposite opening of the bag was secured to a glass tube (1.5 × 4 cm) to facilitate airflow. Bamboo skewers placed into the holding tube and the Y-tube facilitated locomotion of the bioassay male [47]. To initiate a bioassay, an air pump was connected to the holding tube, drawing air at 100 ml/min through the olfactometer. A male that entered the olfactometer within the 5-min bioassay period was classed a responder and two behavioral parameters were recorded: (a) his first choice of oven bag and (b) his engagement, or not, in web-reduction behavior within that bag. Only one of 30 identical olfactometers was deployed for a bioassay at a time and always in the same position. Following a bioassay, the bamboo skewers and bags were discarded, and the glassware was cleaned with soap water and then heated in a drying oven at 100°C for 3 h. In experiments 1–4, 139 males in total were bioassayed, two of which were tested in both experiments 1 and 2, and two other ones in both experiments 1 and 3.

To obtain webs as test stimuli, each randomly chosen virgin female was allowed two days to build her web on an equilateral bamboo frame (8.5 × 8.5 × 8.5 cm) residing in a tray of water [similar to 32]. Thereafter, she was lured to a web-free section of the frame by gentle tapping vibrations and then offered a bamboo stick to walk off the frame on her own accord. This procedure ensured the integrity of her newly-spun web. As webs with or without the female are appealing to males (reviewed in [30]), we opted to test webs on their own.

To obtain a reduced web as a test stimulus, a male was allowed 1 h to enter a web and engage in web reduction. To obtain the wrapped-up section of a web, any visibly bundled-up section was excised from the remainder of that web. To determine whether web reduction by a male renders webs unattractive to rival males, three experiments were run in parallel. Males were given a choice between (a) a frame bearing a female web and an empty frame (Exp. 1, n = 41), (b) a frame bearing an intact female web and a frame bearing a reduced female web (see above) (Exp. 2, n = 41), and (c) a frame bearing the visibly wrapped-up section of a web and a frame bearing the remainder of that same web (Exp. 3, n = 41). Similar numbers of replicates of each experiment were run on the same day, and the position of stimuli in each experiment was randomized. To rule out experimental side bias, males were also offered a choice between two empty frames (Exp. 4, n = 20).

### H2: Deposition of silk by courting males has an inter-sexual signal function that enhances their likelihood of copulation

To test hypothesis 2, the spinnerets of treatment males, but not control males, were rendered non-functional (Exp. 5). Treatment males were anesthetized with CO_2_ and their spinnerets were sealed with super glue gel (LePage, ON, Canada) which was applied under a dissecting microscope via the tip of a 32-gauge silver wire (Bead Landing, TX, USA). CO_2_ -anesthetized control males received the same amount of super glue gel applied to their dorsal abdomen [48]. After glue application, both treatment and control males were given at least 2 h to acclimatize and were then bioassayed within 24 h.

To control for potential effects of male and female size, and mass, on courtship and copulation success, a “condition index” was determined for each spider using regression residuals of the log-transformed body weight and size at maturation (S2 Fig) [49,50]. Prior to testing in experiments, spiders were measured alive. The weight of each male and female was measured on a calibrated scale (Denver Instrument Company TR-204, NY, USA) with an accuracy of 0.1 mg. The size of each male and female was approximated by taking photographs of the first pair of legs under a microscope (Nikon Instruments SMZ1500, NY, USA) with a built-in digital camera (Nikon Instruments DXM1200F, NY, USA), and by measuring the tibia-patella lengths with ImageJ (National Institutes of Health, USA) [50].

For each bioassay replicate (n = 20), two males closely matched in size and condition were assigned to become the treatment or the control male. The two females in each replicate were selected in the same way. The mean percentage difference in the weight and tibia-patella length between male and female pairs were all below 9% (see S1 Table). Females were placed for two days on bamboo frames (30 × 25 × 22 cm) residing in a tray of water to build webs (as in Exps. 1–3).

Each treatment or control male was introduced to the web of a virgin female, residing in a plexiglass box (30.5 × 30.5 × 42 cm) with the female on her web. Courtship was video-recorded for 3 h with two HD cameras (Handycam HDR-XR550; Sony, Tokyo, Japan) under white fluorescent light (2 × 32-watt FO32/835/ECO T8; Sylvania, Wilmington, USA). White-light illumination was chosen to improve the image quality for analyses bearing in mind that *S*. *grossa* females and males do court and copulate under white light [32]. Behavioral elements like web-reduction behavior, latency to copulation, copulation, female aggression (male fleeing in response to female movement) and sexual cannibalism of the male were all determined from the video recordings. As females live up to six years and produce eggs throughout their lives after having copulated once [38,42], we did not quantify the offspring they produced because their lifetime reproductive fitness exceeded the timeframe of this study.

### H3: Stridulatory sound is a courtship signal of males

The stridulatory sound of *S*. *grossa* males in Europe is in the frequency range of 1–7 kHz [34,38]. To test for potential auditory signals produced by courting or copulating males, 20 male-female pairs were video- and sound-recorded, of which 10 pairs each were recorded with a digital sampling rate of 10 kHz and 40 kHz, respectively (Exp. 6). The higher sampling rate (Nyquist frequency) took into account that stridulatory sound of *S*. *grossa* may include frequency components of up to 11 kHz [34]. For each pair, the male and female were randomly selected. The plexiglass box which housed a female web was positioned in the middle of a sound-dampened room and fitted with an AKG CK 61-ULS condenser microphone (AKG Acoustics, Nashville, TN, USA). The microphone was connected to a Dell desktop computer (Dell, Round Rock, TX, USA) equipped with a 16-bit National Instruments (NI) data acquisition card (NI PCIe-6259) (DAQ) and programmed with LabVIEW 7.1 (NI, Austin, TX, USA). The signal-to-noise ratio was improved by pre-amplifying (NI SC-2040 amplifier) potential spider-produced sound prior to digitizing at 10 or 40 kHz via the DAQ card and digitizing the sound on computer [51]. Behavioral elements of males entailing abdominal movements which may produce stridulatory sound [32], namely web jerking (the male vibrating the web with his entire body) (S1 Video and concurrent S1 Audio) and copulation (S2 Video and concurrent S2 Audio), were analyzed for sound including 30 s before they commenced and 30 s after they ended. These paired video and audio recordings were supplemented with audio recordings of background noise in the absence of spiders (S3 Audio) which were then analyzed for sound in the range of 0–5 kHz (sampling rate of recording: 10 kHz) and 1–11 kHz (sampling rate of recording: 40 kHz) using LabVIEW’s Joint Time Frequency Analyzer. To estimate the frequency (Hz) of the males’ abdominal movements during courtship (*sensu* [32]; S1–S3 Videos), high speed video recordings were obtained (Exp. 7). To this end, 12 males were randomly selected and paired with one of 12 females, each on her own web. Abdominal movements of males during courtship were recorded at 30 and 960 frames per second using a Galaxy S9 camera (Samsung, Seoul, South Korea), and were analyzed frame-by-frame using VLC media player (VideoLAN, Paris, France).

Scanning electron micrographs (SEM) of the stridulatory apparatus of male *S*. *grossa* were obtained at the BioImaging Facility of the University of British Columbia (Vancouver, BC, Canada) using a Hitachi S-4700 instrument (Hitachi, Tokyo, Japan). After males were cold-euthanized, their abdomen and prosoma were severed and air-dried for 48 h. Both tagmata were then mounted with double-sided carbon tape on aluminum pin stubs at an angle most conducive for viewing of the stridulatory apparatus. After sputter-coating both tagmata with a 15-nm thick layer of gold using the rotary-planetary-tilting stage of a Cressington 208HR instrument at a 60-mA current, SEMs were taken using various imaging modes and accelerating voltage.

### Statistical analyses

Data were analyzed with R [52,53]. In experiments 1–4, first-choice responses were analyzed by χ^2^-tests against an expected frequency of 50:50, whereas the proportion of males that engaged in web reduction in response to either of the two presented test stimuli was compared with a χ^2^-test. Data of experiment 5, which tested the effect of spinneret occlusion on the occurrence of specific behavior (web reduction, copulation, cannibalism), were analyzed with either a generalized linear model (GLM) or a generalized linear mixed model (GLMM), with spinneret treatment included as the sole fixed effect. Mixed effect models [54] incorporated the effect of treatment and control male pairs into the models as a random intercept. If variance in these intercepts approached 0, mixed models were abandoned in favor of a simple χ^2^-test. We also analyzed the effect of duration of web-reduction behavior on the latency to copulation and the occurrence of copulation and cannibalism by females with GLMs or GLMMs, with duration as the sole fixed effect.

## Results

### H1: Web reduction by males renders webs less attractive to rival males (Exps. 1–4)

**Fig 1:**
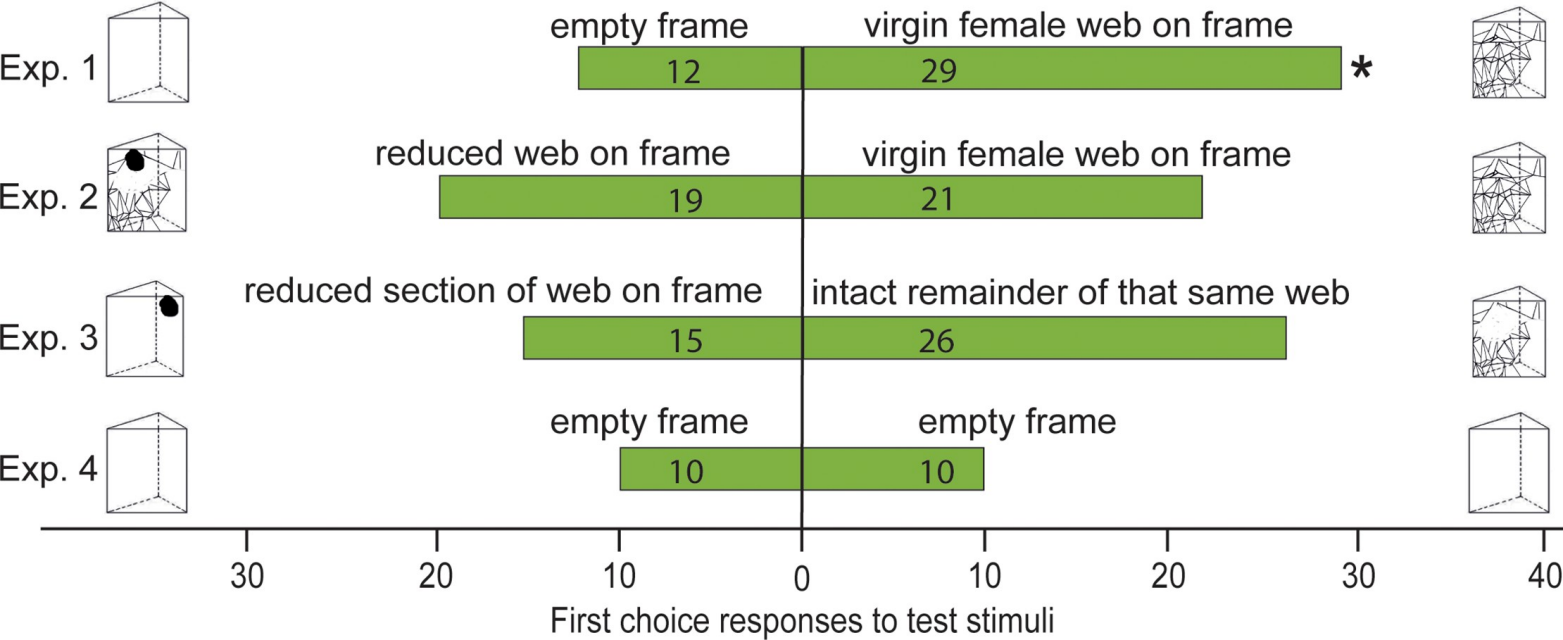
Anemotactic attraction of male *Steatoda grossa*. First-choice responses of males to specific test stimuli in Y-tube olfactometer experiments 1 (n = 41), 2 (n = 40), 3 (n = 41) and 4 (n = 20). Numbers in bars indicate the number of males choosing the respective stimulus. One male did not respond in Exp. 2. The asterisk (*) denotes a significant preference for the respective stimulus; χ^2^ test; p < 0.05.

Males significantly more often entered first those oven bags that enclosed a frame with an intact web than oven bags enclosing an empty control frame (χ^2^ = 7.05, df = 1, n = 41, p < 0.01; Fig 1, Exp. 1). In contrast, oven bags enclosing a frame with an intact web or a frame with a reduced web were entered first equally by males (χ^2^ = 0.10, df = 1, n = 40, p = 0.752; Fig 1, Exp. 2). Similarly, oven bags enclosing a frame with the excised wrapped-up section of a web or a frame with the corresponding intact remainder of that same web were entered first equally often by males (χ^2^ = 2.95, df = 1, n = 41, p = 0.086; Fig 1, Exp 3). No experimental side bias of males was observed (χ^2^ = 0, df = 1, n = 20, p = 0.5; Fig 1).

**Fig 2:**
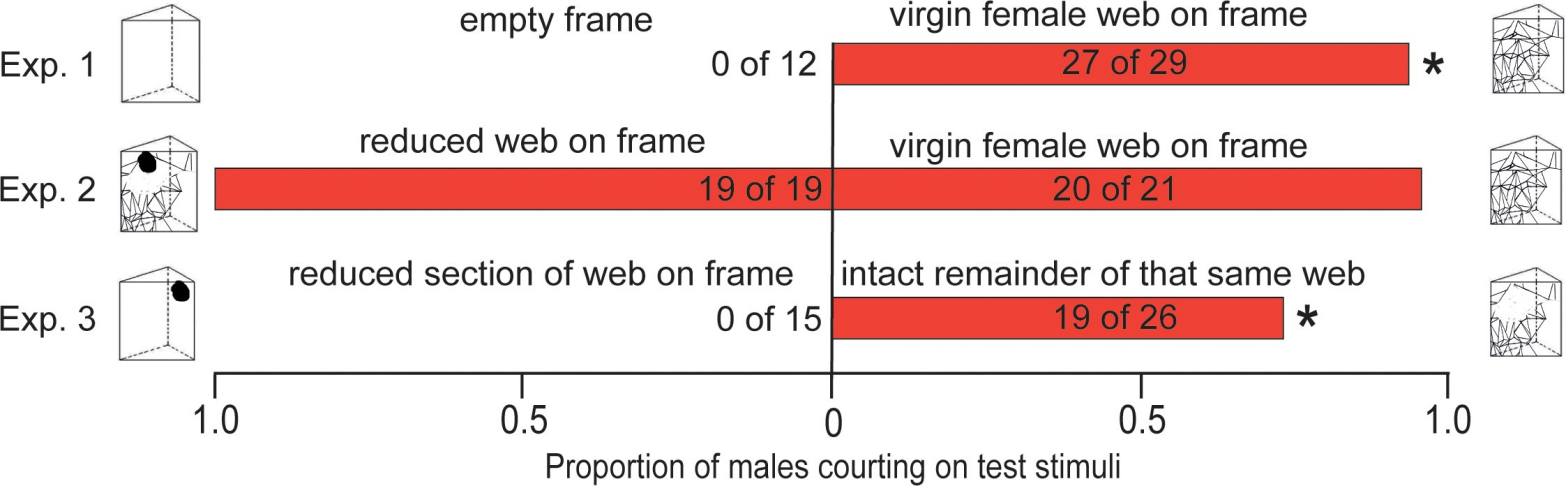
Occurence of web reduction by male *Steatoda grossa*. Proportion of *Steatoda grossa* males engaging in web-reduction behavior (element of courtship display) in response to test stimuli. In each of experiments 1 (n = 41), 2 (n = 40), and 3 (n = 41), the asterisk (*) denotes a significant preference for the respective stimulus; χ^2^ test; p < 0.05.

Males engaged in web-reduction behavior only on frames bearing an intact web but not on empty control frames (χ^2^ = 32.72, df = 1, n = 41, p < 0.001; Fig 2, Exp. 1). A similar proportion of males web-reduced on frames bearing an intact web and on frames bearing a reduced web (χ^2^ = 0.89, df = 1, n = 40, p = 0.345; Fig 2, Exp. 2). However, when offered a choice between frames bearing the excised wrapped-up section of a web or the remainder intact section of that same web, males engaged in web-reduction behavior only on the intact remainder of the web (χ^2^ = 20.43, df = 1, n = 41, p < 0.001; Fig 2, Exp. 3).

### H2: Deposition of silk by courting males has an inter-sexual signal function that enhances their likelihood of copulation (Exp. 5)

Fourteen out of 40 female-male pairs copulated. Ten of these 14 pairs involved males with functional spinnerets, making them more likely to copulate than males with dysfunctional spinnerets (Table 1). The functionality of the males’ spinnerets had an effect on (*i*) the time males spent web-reducing (Table 1), but not on the latency to copulation (Table 1) and the time spent in copula (Table 1). Most webreduction behavior and most copulations occurred on the sheet area [55] of the webs.

**Table 1:** Dysfunctional spinnerets affect courtship behavior and copulation success of male *Steatoda grossa*.

	Spinnerets		
Criteria recorded	Dysfunctional	Functional	Statistical results
Copulations	4	10	χ^2^ = 4.72, df = 1, p = 0.03
Web-reducing	17	20	χ^2^ = 3.24, df = 1, p = 0.07
Mean ± SE time males spent web-reducing	346 ± 119 s	899 ± 149 s	χ^2^ = 10.91, df = 1, p = 0.001
Mean ± SE latency to copulation	3311 ± 1943 s	4989 ± 2299 s	F_1,13_ = 0.53, p = 0.48
Mean ± SE duration of copulation	1298 ± 577 s	1000 ± 682 s	F_1,13_ = 0.19, p = 0.67
Female cannibalism	1	1	χ^2^ = 0.00, df = 1, p = 1

Criteria recorded during courtship of 40 male-female *Steatoda grossa* pairs, with 20 males having dysfunctional (experimentally occluded) spinnerets and 20 males having functional (intact) spinnerets. The table shows either the number of occurrences or the mean ± standard error, together with significance testing of the spinneret treatment effect (from generalized mixed models). The calculated means for latency to, and duration of, copulation were calculated only for males that copulated, whereas means of web-reduction behavior include data from all males.

**Fig 3:**
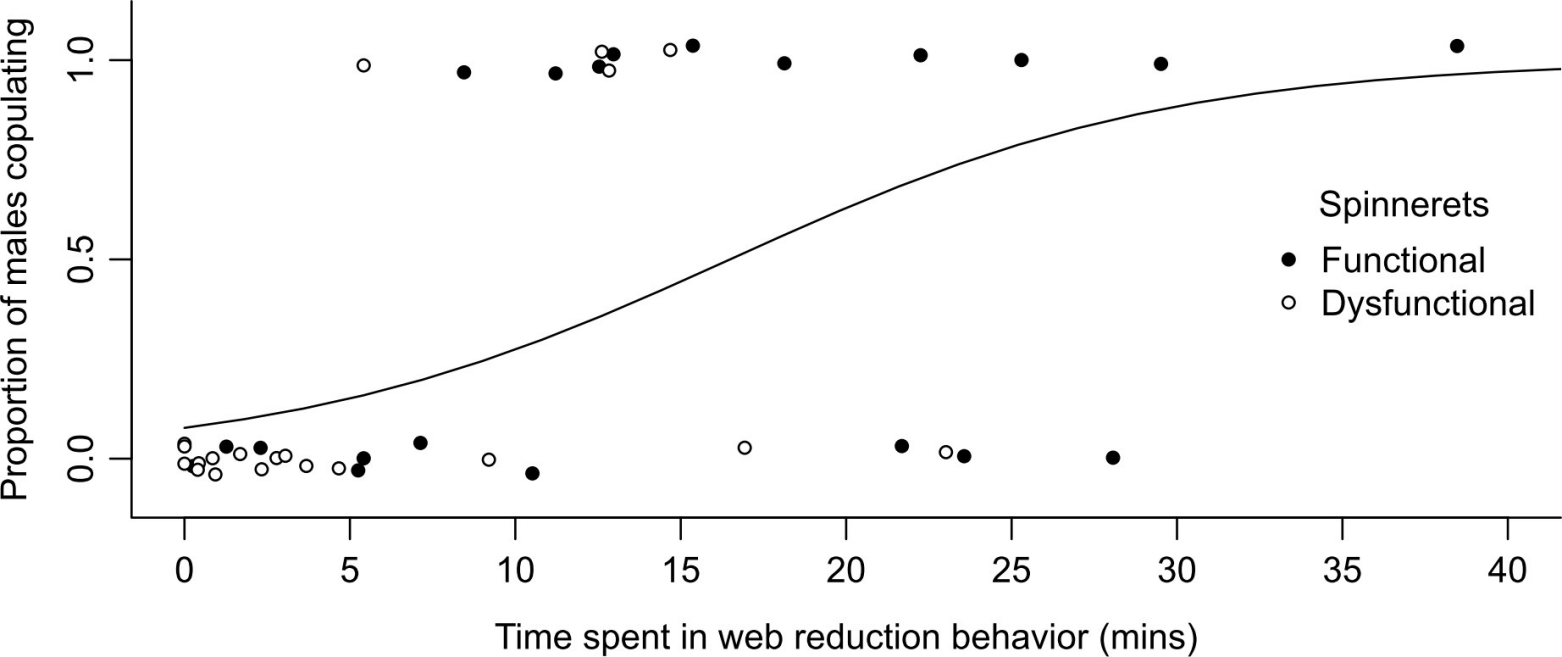
Web reduction by male *Steatoda grossa* and likelihood of copulation. The likelihood of males with functional or dysfunctional spinnerets to copulate with the female they courted increased with increasing time they engaged in web-reduction behavior; general linear mixed model, p = 0.0009; the line shows the predicted likelihood of copulation in relation to the time spent in web reduction.

The likelihood of males with functional or dysfunction spinnerets to copulate with the female they courted increased with increasing time they spent web-reducing (χ^2^ = 10.97, df = 1, p = 0.0009; Fig 3, Exp. 5). However, the time males spent web-reducing had no effect on the latency to copulation (F_1,13_ = 0.67, p = 0.43; Fig 3, Exp. 5).

Female aggression towards males was not affected by the time males spent web-reducing (χ^2^ = 0.37, df = 1, p = 0.54). One male with functional spinnerets and one male with dysfunctional spinnerets were cannibalized by the courted female.

### H3: Stridulatory sound is a courtship signal of males (Exps. 6–7)

**Fig 4:**
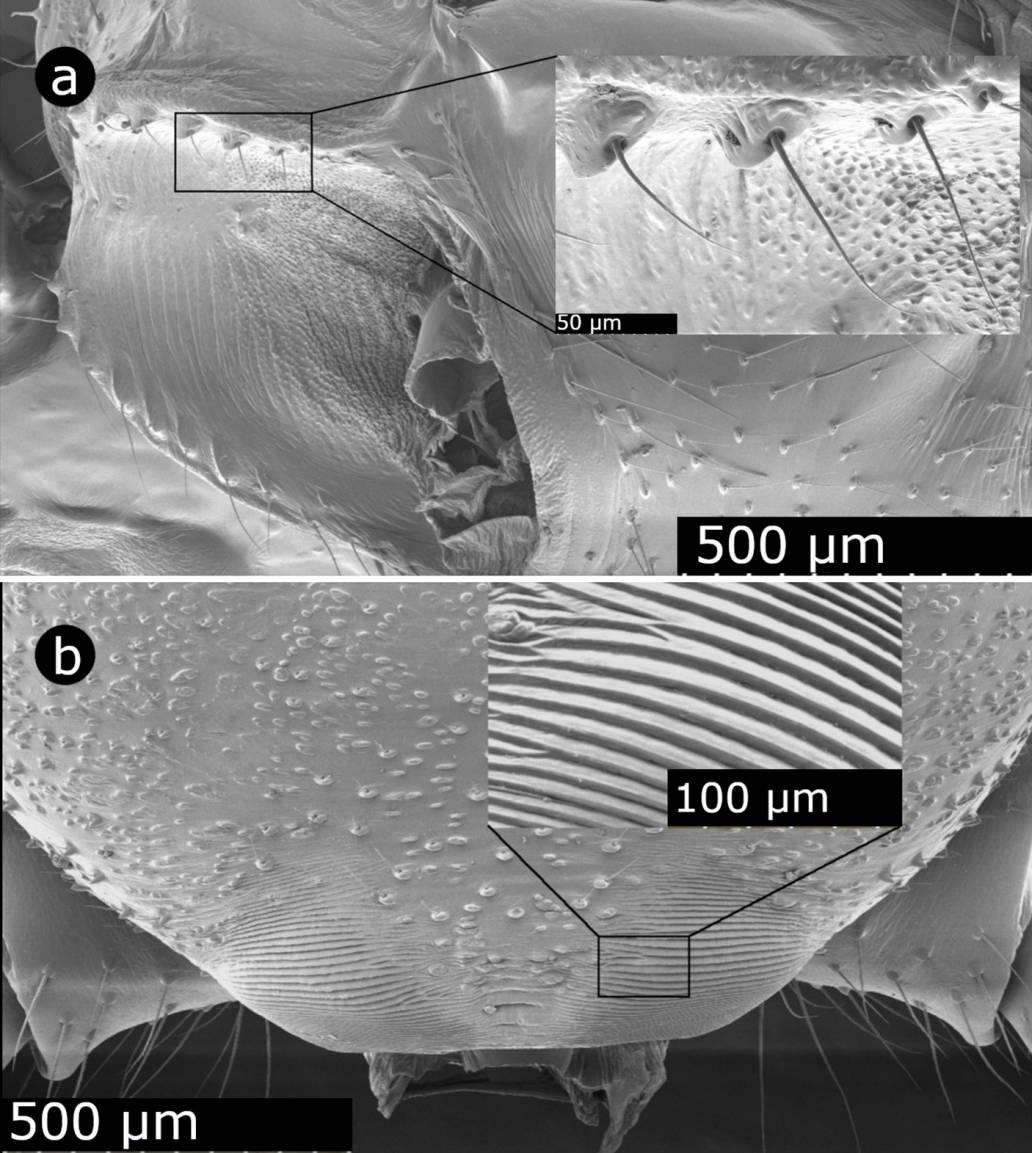
Stridulatory apparatus of a male *Steatoda grossa*. Scanning electron micrographs show (a) teeth-like structures (the scraper) on the anterior ventrum of the abdomen, and (b) ridges (the file) on the posterior tergum of the prosoma (cephalothorax).

The stridulatory apparatus of *S*. *grossa* males in a North American population closely resembled that of males in a European population [38,40]. SEM images of males from North America revealed tooth-like structures on the abdomen (Fig 4A) that could be scraped over ridges on the prosoma (Fig 4B), thus producing sound.

The mean (± SE) time for the up- and down-movement of the male’s abdomen was 0.195 ±0.011 s and 0.219 ± 0.026 s, respectively (Exp. 7, S3 Video). Sound recordings during abdominal movements of males when stridulatory sound may occur (S1 Video, S1 Audio; S2 Video, S2 Audio) revealed no sound in the frequency range of 0–11 kHz that could have served as courtship signals (S1B Fig). Background noise sound recordings (see S3 Audio as an example) indicated frequency components in the range of 0.5–1.5 kHz, 3–4 kHz and 7 kHz at very low levels and entirely dissimilar to the stridulatory sound produced by European *S*. *grossa* males (S1A Fig). Thirteen out of 20 female-male pairs recorded in the context of H3 copulated, and no male was cannibalized.

## Discussion

We present data indicating that web reduction behavior by *Steatoda grossa* males in North America has no long-range (*sensu* [56]) effect on mate-seeking females. Instead, web-reduction behavior has an inter-sexual (male-female) signaling function that appears to be linked to functional spinnerets of the courting male. The inter-sexual signaling function seems to be based on a silk-borne pheromonal signal produced by the courting male but may also be modulated by the amount of time males engage in web reduction causing web vibrations. Males did not produce any stridulatory sound during courtship or copulation, although they possess the stridulatory apparatus for sound production. Below, we elaborate on these conclusions, using the three hypotheses as subheadings.

### H1: Web reduction by males renders webs less attractive to rival males

Webs of *S*. *grossa* females reduced by a courting male were as attractive to other males as intact webs, indicating that web-reduction behavior has no long-range effect on mate-seeking males. Moreover, the intact section of reduced webs continued to prompt web reduction by late-arriving males. These results are surprising considering the first sperm precedence in the entelegyne *S*. *grossa* [34,57]. To recognize a reduced web from a distance would be adaptive for mate-seeking males as they would save energy avoiding webs of a female that has already mated. In *L*. *hesperus*, reduced webs are indeed significantly less attractive to males than intact webs [13]. As *L*. *hesperus* and *S*. *grossa* males exhibit the same type of web-reduction behavior and achieve comparable results in the form of reduced webs, it is perplexing that reduced *S*. *grossa* webs remain attractive to males. It seems that web-reducing behavior of *S*. *grossa* males may have only recently evolved [32,33,38], and that this behavior has not yet curtailed long-range attraction of mates, as it has in *L*. *hesperus* [13]. Evidence for an evolving communication system in *S*. *grossa* stems from five studies with spiders originating from the same geographic location in Europe. Early (past-century) studies [36,38,39] report stridulatory courtship of males without web-reducing behavior, whereas more recent (2004, 2018) studies [33,34] report both stridulatory sound and web reduction.

Our observations that *S*. *grossa* males did not reduce already reduced sections of a web imply that they sensed male pheromone on male silk upon contact, male silk impeded access to female silk bearing female contact sex pheromone, or both.

### H2: Deposition of silk by courting males has an inter-sexual signal function that enhances their likelihood of copulation

The differential success of males with functional and dysfunctional spinnerets in securing a copulation with the female they courted suggests that male silk and silk-borne pheromone, respectively, serves as a male-female sexual communication signal. Web reducing has previously been recognized as an essential element of courtship behavior of (false) black widow males that affects their likelihood of copulating with the courted female [30,32, this study]. However, the relative contributions of the vibratory signals associated with the cutting of the female’s web and the male’s silk used to bundle the cut-up sections, remained unknown. As males with functional and dysfunctional spinnerets exhibited visually comparable web-reduction behavior, but mostly the males that could disseminate silk secured copulations, it follows that male silk enhances a female’s receptivity. However, because males with functional spinnerets spent more time reducing and thus vibrating webs than males with dysfunctional spinnerets, it is still conceivable that both male silk and the extend of web vibrations affect the female’s receptivity and the male’s likelihood of copulation with the courted female. As not every female that eventually copulated made physical contact with the male’s silk, it follows that it is likely a silk-borne volatile male pheromone that–alone or in combination with vibratory signals–renders the female receptive. Given the rather small amount of silk that the male deposits on a female’s web during courtship, it is not surprising that only a single male pheromone (*Z*-9-tricosene) has been identified to date [58].

Curiously, the ability to, or not to, deposit silk during courtship had no effect on other aspects of courtship behavior and interactions between the female and the male, such as cannibalism of males by females, the latency to copulation and the copulation duration. Males with the superglue control treatment (Exp. 5), and those without superglue application (Exp. 6), secured similar numbers of copulations (10 of 20 and 13 of 20 pairs, respectively), indicating that superglue had no adverse effects on the courtship success of males.

### H3: Stridulatory sound is a courtship signal of males

Male stridulatory signals are part of the courtship repertoire in European populations of *S*. *grossa* [33,38] but were absent in a North American population of *S*. *grossa* (this study). Sound recordings revealed no evidence that courting or copulating males produce sound (S1 Fig) remotely resembling that previously reported for *S*. *grossa* males in a European population (S1 Fig) [38]. Abdominal movements of males that seemed suggestive of causing stridulatory sound [32] were too slow (by 2 orders of magnitude), and the angle of movements seemed too shallow [see 38], to produce stridulatory courtship sound resembling that of *S*. *grossa* males in a European population [38]. Males “being silent” during courtship in our study may explain the relatively low number of copulations (27 out of 60 pairs) that were observed. If females still anticipated stridulatory sound from courting males, then all these silent males would have been appraised inferior prospective mates. It would be intriguing now to study the incidence of copulation in an experiment with a full factorial design, each replicate involving four male-female pairs: two pairs with both the female and the male originating from the same population in Europe or in North America, and two pairs with the female or the male selected from the European or the North American population.

The reason why *S*. *grossa* males from the North American population we studied here are silent, not using their stridulatory apparatus during courtship, is unclear. Typically, courtship sound is selected against when it draws the attention of potential predators, as shown in crickets [59], but there is no prevalent predator of *S*. *grossa*, or other spiders, in North America known to exploit spider courtship sound as a prey location cue. There is also no apparent selection pressure for the evolution of sound as an honest male courtship signal because sound production requires minimal nutritional energy and thus, unlike silk production and deposition by males, is not indicative of a male’s physical condition [23,24]. However, there may be selection pressure for male *S*. *grossa* to shift from sound to chemical communication during courtship in the noisy urban soundscape that *S*. *grossa* typically inhabits. That courting *S*. *grossa* males exclusively deposited silk (chemical communication) or stridulated (sound communication) [33] supports the concept that this type of shift in communication modality may well be under way. Alternatively, a “genetic bottleneck” in the invaded North American range could have prompted a shift in courtship signaling, especially if stridulation is encoded by a few major loci rather than many minor loci [60]. Such a bottleneck can be expected in the noisy urban soundscape inhabited by *S*. *grossa* [19–22].

## Conclusion

In the invaded North American range, web-reduction behavior by *S*. *grossa* males has no long-range effect on mate-seeking males. For these males, it would be adaptive to avoid reduced webs occupied by mated females. Yet, reduced webs remained as attractive as intact webs occupied by virgin females, implying that web-reduction behavior by males has only recently evolved and that a “reduced mate-competition” function of this behavior is not yet established. However, web-reduction behavior by *S*. *grossa* males does have an inter-sexual (male-female) signaling function that appears to be linked to functional spinnerets of the courting male. The inter-sexual signaling function seems to be based on a silk-borne pheromonal signal produced by the courting male but may also be shaped by the extent of time during which web vibrations caused by web reduction occur. Males did not produce any stridulatory sound during courtship or copulation, although they possess the stridulatory apparatus for sound production.

## Supporting information

S1 Fig Courtship sound of male *Steatoda grossa*. Representative recordings of stridulatory sound produced by courting *S*. *grossa* males from a European population (a) and a North American population (b). Graphs show the sonogram, power spectrum and waveform of recorded stridulatory sound. Note the absence of any sound signal produced by the male in (b); The recording in (a) was made available by Rainer Welzenberger [34].
(TIF)

S2 Fig Regression of tibia-patella length against natural-log-transformed body weight of male and female *Steatoda grossa*. This regression was used to calculate a condition index for matching experimental pairs in experiment 5. Regression models were fit using a linear model (females) or a linear mixed model (males). A mixed model was used to fit a different slope to each of three manually identified tibia-patella length ranges. Sub-adult males were kept separate from virgin females, because volatile sex pheromone components of females induce maturation rather than growth of males. When the number of adult males became limited, we moved sub-adult males into the same room where we kept virgin females. This prompted maturation of smaller-sized males, necessitating the use of different condition indices.
(TIF)

S1 Table Summary of percentage differences between pairs of male and female *Steatoda grossa*. In experiment 5, male and female pairs were matched in size and condition (see S2 Fig). The table displays the mean, standard deviation, and maximum percentage difference in weight and size between paired spiders.
(CSV)

S1 Video Web jerking behavior of male *Steatoda grossa* during courtship. The video recording (pertinent to Exp. 6), shows a courting male and a female on a web built on a wooden frame. The microphone (visible in the upper left section of the footage) recorded potential stridulatory sound (S1 Audio) during abdominal movements of the male which are visible between web-jerks.
(MP4)

S2 Video Copulation of male and female *Steatoda grossa*. The video recording (pertinent to Exp. 6) shows a male and a female *in copula* on a web built on a wooden frame. The microphone (visible in the upper left section of the footage) recorded potential stridulatory sound (S2 Audio) during abdominal movements of the male.
(MP4)

S3 Video High speed video recording of abdominal movements by a male *Steatoda grossa*. The recording shows a courting male exhibiting abdominal movements. The first three seconds of the file were recorded with 30 frames per second (fps) and are shown at 7.5 fps. The following six seconds were recorded in slow motion (960 fps) and are shown at 29 fps (near normal frequency).
(MP4)

S1 Audio Web jerking behavior of male *Steatoda grossa* during courtship. This audio recording was concurrently obtained with the video recording (see S1 Video). The audio file revealed no evidence for stridulatory courtship sound. The audio file was generated by playback of the recorded log file and was re-recorded as a Wavefrom Audio File (wav) because conversion software is not available.
(WAV)

S2 Audio Copulation of male and female *Steatoda grossa*. This audio recording was concurrently obtained with the video recording (see S2 Video). The audio file revealed no evidence for stridulatory sound during copulation. The audio file was generated by playback of the recorded log file and was re-recorded as a Wavefrom Audio File (wav) because conversion software is not available.
(WAV)

S3 Audio Background recording in the absence of spiders. This audio recording was obtained prior to introduction of spiders for S1 and S2 Audio. The audio file was generated by playback of the recorded log file and was re-recorded as a Wavefrom Audio File (wav) because conversion software is not available.
(WAV)
