# Supplementary figures and images for: Multimodal and multifunctional signaling? – Web reduction courtship behavior in a North American population of the false black widow spider
Source: PLoS One. 2020 Feb 26;15(2):e0228988. doi: 10.1371/journal.pone.0228988 (PMC7043733; doi:10.1371/journal.pone.0228988)

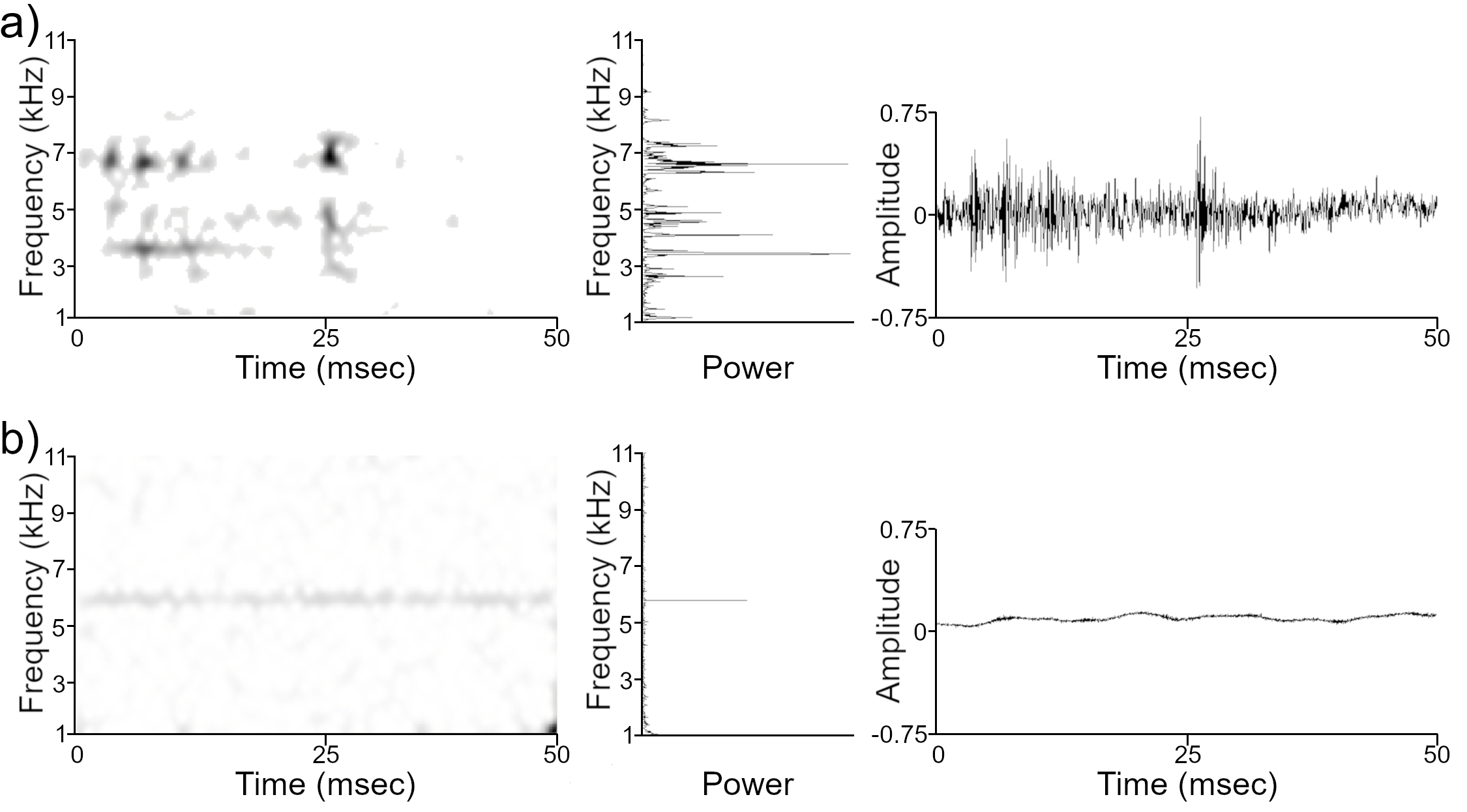

Supplement: S1 Fig — Representative recordings of stridulatory sound produced by courting S. grossa males from a European population (a) and a North American population (b). Graphs show the sonogram, power spectrum and waveform of recorded stridulatory sound. Note the absence of any sound signal produced by the male in (b); The recording in (a) was made available by Rainer Welzenberger [34]. (TIF) [file pone.0228988.s001.tif]

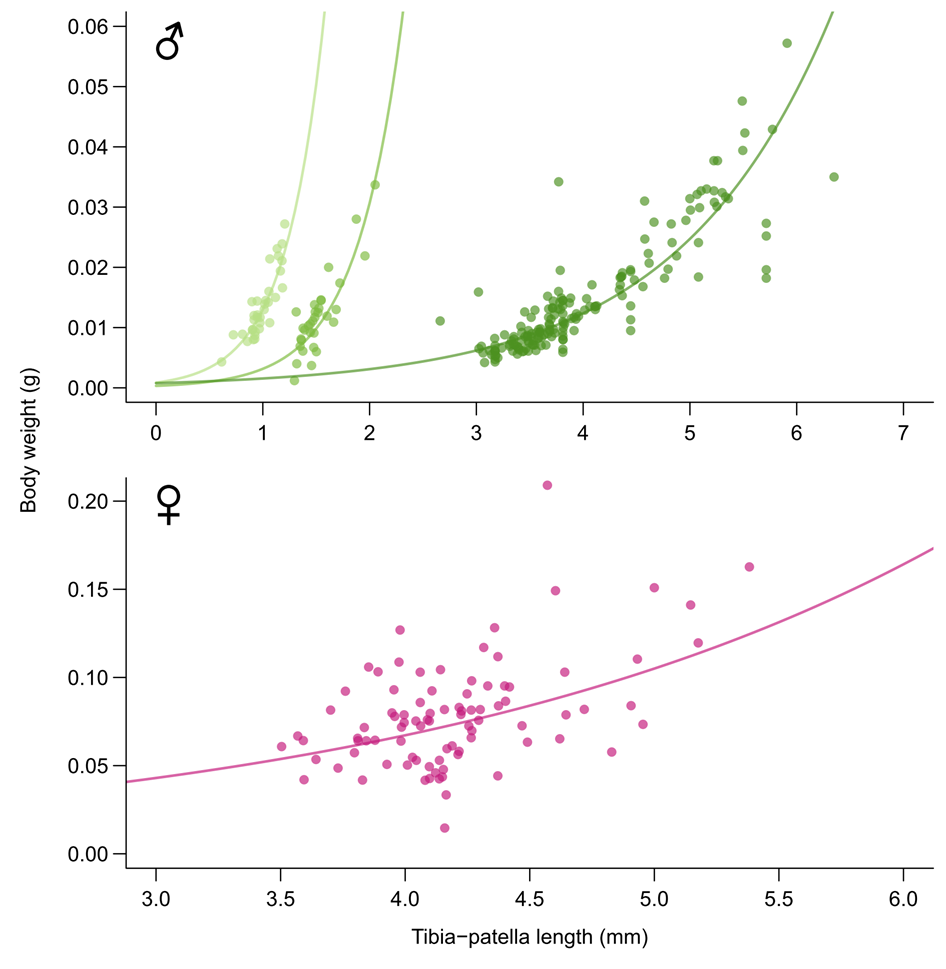

Supplement: S2 Fig — This regression was used to calculate a condition index for matching experimental pairs in experiment 5. Regression models were fit using a linear model (females) or a linear mixed model (males). A mixed model was used to fit a different slope to each of three manually identified tibia-patella length ranges. Sub-adult males were kept separate from virgin females, because volatile sex pheromone components of females induce maturation rather than growth of males. When the number of adult males became limited, we moved sub-adult males into the same room where we kept virgin females. This prompted maturation of smaller-sized males, necessitating the use of different condition indices. (TIF) [file pone.0228988.s002.tif]
